# Internet-based cognitive behavioral therapy for psychological symptoms during the final phase of the COVID-19 pandemic: a feasibility study

**DOI:** 10.3389/fdgth.2025.1504217

**Published:** 2025-06-09

**Authors:** Victoria Aminoff, My Björklund, Elina Ekström, Andrea Stenback, Uzma Yousafzai, Matilda Berg, Mikael Ludvigsson, Gerhard Andersson

**Affiliations:** ^1^ Department of Behavioural Sciences and Learning, Linköping University, Linköping, Sweden; ^2^ Department of Biomedical and Clinical Sciences, Linköping University, Linköping, Sweden; ^3^ Department of Psychiatry in Linköping, Department of Biomedical and Clinical Sciences, Linköping University, Linköping, Sweden; ^4^ Department of Geriatrics and Palliative Medicine, Department of Health, Medicine and Caring Sciences, Linköping University, Linköping, Sweden; ^5^ Department of Clinical Neuroscience, Karolinska Institute, Stockholm, Sweden

**Keywords:** COVID-19, internet-based cognitive behavioral therapy, psychological treatment, post-COVID, feasibility study

## Abstract

**Introduction:**

SARS-CoV-2 and the accompanying COVID-19 pandemic had a great impact on people's well-being, both physically and mentally. The pandemic continued to affect people even after its end was declared. Internet-based cognitive behavioral therapy (ICBT) is a psychological treatment alternative that is effective for several types of psychological symptoms and conditions. This study aimed to investigate the feasibility of ICBT for psychological symptoms related to the COVID-19 pandemic in adults aged 18 years and older during the final phase of the pandemic. Since the psychological impact of the pandemic varies among individuals, individually tailored ICBT was examined, in which participants receive different treatment content based on their needs.

**Methods:**

A within-group study was conducted with 24 participants, receiving individually tailored ICBT during eight weeks with weekly support from a therapist. Participants received one module per week, which was selected based on the participant's specific symptoms and needs. Of the 24 participants, 16 (66.7%) were assessed as probably meeting the diagnostic criteria for post-COVID syndrome. Pre- and post-treatment measurements using self-report questionnaires for several psychological symptoms were administered online.

**Results:**

Statistically significant improvements were observed in symptoms of depression, anxiety, post-traumatic stress, loneliness, and exhaustion. Small increases were observed in a measure of CBT knowledge, whereas no significant changes were found in stress, quality of life, experience of cognitive failures, and economic stress. Participants completed on average 3.96 out of 8 modules, with five (20.8%) completing all modules. Fifteen (62.5%) of the 24 participants completed the post-treatment measurement. Three participants (12.5%) withdrew from the study.

**Conclusion:**

Overall, the results indicate that individually tailored ICBT with weekly support from a therapist is a feasible treatment for psychological symptoms related to the COVID-19 pandemic in its final phase. However, larger studies with more participants are needed to draw further conclusions regarding the effects of ICBT during the final phase of a pandemic. The treatment could be further developed to benefit a broader range of participants.

## 1 Introduction

The COVID-19 pandemic, with the spread of the SARS-CoV-2 virus, has considerably impacted individuals' physical and mental well-being (1–3). The SARS-CoV-2, causing the illness coronavirus disease 2019 (COVID-19), can result in somatic symptoms such as cough, sore throat, fever, and myalgia (4), and in severe cases result in death (5). Mental well-being can be affected due to factors such as fear of infection, fear of dying, and a sense of helplessness (3), as well as by spread-related restrictions such as isolation and quarantine (6, 7).

When the incidence of psychological symptoms was investigated during initial (during the year 2020) and middle (during the year 2021) phases of the COVID-19 pandemic, the prevalence rates for depression, anxiety, and stress were found to be around 30% (3, 8). These prevalences can be compared with the 12-month prevalence reported before the COVID-19 pandemic, which was 6.9% for depression and 14% for anxiety (9), indicating that symptom levels did increase during the pandemic. Consistent with these findings, Pieh et al. (10) reported elevated levels of depressive, anxiety, and insomnia symptoms during the pandemic relative to pre-pandemic baseline levels. However, it is difficult to estimate how and to what extent individuals' mental well-being was affected during the pandemic. In their systematic review, Alqahtani et al. (1) concluded that depression, anxiety, and stress increased during the COVID-19 pandemic, but that the prevalence of these three conditions varied depending on region and other factors. For example, the prevalence of depression reported in the studies reviewed by Alqahtani et al. (1) ranged from 14.6% to 45%. For anxiety, the prevalence ranged from 8.3% to 47% (1). This heterogeneity is in line with earlier research regarding the impact on individual mental health during former phases of the COVID-19 pandemic. For instance, Mahmud et al. (11) showed that the prevalence of various psychological symptoms and conditions differed between studies focusing on various populations, regions, and time periods. Overall, it can be stated that individual mental health was adversely affected to some extent (1, 12), even among those who were not infected with the SARS-CoV-2 virus (13). Elevated levels of psychological symptoms have furthermore been reported to persist even in the later phases of the COVID-19 pandemic, when societal restrictions imposed due to the pandemic had been largely lifted (14).

Deng et al. (15) investigated mental health in those infected by the SARS-CoV-2 in a systematic review. They reported pooled prevalence estimates for depression, anxiety, and sleep disturbance of 45%, 47%, and 34%, respectively. No gender differences were found (15). A considerable number of people who have recovered from the acute phase of COVID-19 experience persistent symptoms that impair their daily activities beyond the initial illness (16, 17). This has led to the description of a post-COVID syndrome, defined as symptoms that develop during or after a COVID-19 infection, persisting for more than twelve weeks, and cannot be attributed to another diagnosis (18). Common symptoms reported are fatigue, dyspnea, sleep disorder (19), as well as cognitive dysfunctions such as concentrating difficulties, short-term and general memory loss, and impairment of executive functions (20). As with the general population during the COVID-19 pandemic and those being infected by the SARS-CoV-2, a significant number of post-COVID syndrome patients also report psychological symptoms, such as depression, anxiety, and post-traumatic stress disorder (20).

Internet-based cognitive behavioral therapy (ICBT) has been investigated as an alternative, or complement, to CBT face-to-face (21). ICBT is cost-effective when including weekly support by a therapist (22), not requiring as much therapist time as CBT face-to-face. At the same time, ICBT, when therapist support is included, has been shown to yield effects comparable to face-to-face CBT (23). There is also evidence supporting the effectiveness and acceptability of ICBT in routine clinical settings (24). ICBT can be delivered in various formats, such as with or without therapist guidance, and as either a standardized program applied uniformly to all participants or as an individually tailored intervention. In many studies, therapist-supported ICBT has demonstrated superior outcomes compared to unsupported formats (25), although self-guided treatments also have shown to yield clinically significant effects (26). Tailored ICBT, in which modules are chosen depending on the individual's specific symptoms and situation, has been found to be effective for depression and anxiety symptoms, as well as quality of life (27, 28). The treatment may consist of a selection of so-called treatment modules, which commonly address various typical CBT strategies such as behavioral activation, exposure, and emotion regulation, and can also focus on different areas, such as stress or perfectionism (27). The modules are typically text-based, with associated exercises, but may also include explanatory images or videos (29). With an individually tailored intervention, different sets of symptoms and comorbidity can be addressed (30). This is because the individually tailored approach allows individuals to undergo the same overall treatment program while receiving different content based on their psychological needs and symptoms. In contrast to non-tailored ICBT, where all participants work through the same treatment material, the tailored format offers flexibility by allowing the treatment components to be assembled in a modular fashion. Thus, rather than delivering a fixed set of treatment modules to all individuals, different modules can be provided in varying sequences depending on individual needs (27). People have been affected by the COVID-19 pandemic in different ways (1), which motivates an individually tailored ICBT approach.

During the COVID-19 pandemic, several trials were conducted investigating the effects of ICBT. Komariah et al. (31) examined the effects of ICBT during the pandemic in a systematic review and meta-analysis, including nine randomized controlled trials (RCTs). The trials varied in treatment format, including the focus of the intervention and the type and frequency of therapist support. Even if all studies targeted psychological symptoms related to the COVID-19 pandemic, the trials varied somewhat in terms of psychological symptom focus, e.g., depression and anxiety (32), only worry (33), only depression (34), and health anxiety (35). All trials shared the same target group, namely adults experiencing psychological symptoms related to the COVID-19 pandemic, except for one study (36), targeting COVID-19 patients. Aggregated results demonstrated ICBT as effective in reducing depression and anxiety symptoms during the COVID-19 pandemic, even if the studies differ in many aspects. Therapist-guided ICBT interventions included in the meta-analysis were shown to be superior to self-guided regarding the decrease in depression symptoms from pre- to post-treatment measurement (31). These results align with results displayed before the COVID-19 pandemic, showing that ICBT with therapist support is more effective than ICBT without (37). Regarding anxiety symptoms, however, the results were the opposite, showing self-guided ICBTs being superior to therapist-guided ones (31). When Mahoney et al. (38) investigated the effects of ICBT on symptoms of depression and anxiety before and during the initial and middle phases of the COVID-19 pandemic, no differences were shown, and thus, they concluded that ICBT remains effective in a pandemic context.

Additional studies on ICBT have been conducted during the COVID-19 pandemic, with somewhat conflicting results compared to those reported by Komariah et al. (31). For example, no significant effects on symptoms of depression and anxiety were found when Brog et al. (39) investigated a three-week-long internet-based self-help intervention aiming to target psychological distress related to the COVID-19 pandemic. These results differ from those found by Wahlund et al. (33), when they investigated a three-week-long self-guided treatment. Significant reductions in worry related to the COVID-19 pandemic and depression symptoms were shown in their study (33). Overall, trials investigating ICBT during the COVID-19 pandemic indicate that ICBT has potential. Still, the results differ to some extent (e.g., 33, 39), implying a need for further research within this area. Also, to the best of our knowledge, most of the ICBT trials have been conducted in the early phases of the COVID-19 pandemic (e.g., 33, 36, 40, 41), with only a few carried out during its mid-phase (e.g., 42, 43). During the pandemic's final phase, many people were vaccinated (44) and had been infected by SARS-CoV-2 at some point, resulting in a lower risk of somatic illness in case of infection and spreading of the virus in total and, thus, fewer restrictions prevailed than in the pandemic's former phases. However, this does not necessarily mean that the impact on the mental health diminishes (14). While many had the opportunity to return to a somewhat “normal” state, the pandemic's aftermath, for instance in terms of social contacts, was still current for some people. Thus, a need for psychological interventions in relation to the pandemic could still exist. Although other forms of internet-based psychological treatments exist and have been shown to be effective for depressive and anxiety symptoms among others, such as psychodynamic (45) or interpersonal (46) interventions, it is to our knowledge internet-based interventions with a CBT approach that have been most extensively studied during the COVID-19 pandemic. Thus, we aim to expand this area of research. Whether ICBT would be effective and feasible during the pandemic's final phase, as in its early and middle phases, is still relatively unexplored.

Taken together, the mental health during the COVID-19 pandemic has worsened in different areas (1), both in people infected by the SARS-CoV-2 (15) and not (13). ICBT has been investigated but, to our knowledge, not during the final phase of the pandemic. This study aimed to examine the feasibility of individually tailored ICBT with weekly support by a therapist during the final phase of the COVID-19 pandemic. The pandemic was still ongoing during this period, but many people were slowly transitioning back to “normal”, i.e., returning to their everyday life and lifestyle before the outbreak. At the same time, psychological symptom levels remained elevated relative to pre-pandemic baselines (14), and not all had the opportunity to go back to a “normal”, pre-pandemic, state. Thus, we aimed to evaluate whether ICBT, a psychological treatment that does not increase the risk of infection spread, can be effective for psychological symptoms that emerged or were exacerbated in relation to the COVID-19 pandemic and its consequences.

## 2 Methods

### 2.1 Study design

A within-group design was used to investigate whether individually tailored ICBT with weekly therapist support would be a feasible treatment alternative for psychological symptoms related to the COVID-19 pandemic in its final phase. Data were collected using online surveys, comprising several questionnaires, administered before and after the eight-week long treatment. The study was originally intended to be a randomized controlled trial (RCT). Due to the small number of participants, a within-group design was used instead, primarily evaluating the change in participants' self-rated levels of depression and anxiety between pre- and post-treatment measurement. The trial was registered on ClinicalTrials.gov (NCT05656430) and approved by The Swedish National Ethics Committee (Dnr 2022-05268-01).

### 2.2 Procedure

#### 2.2.1 Recruitment

Registration for the study was open from mid-January 2023 until three weeks later. Recruitment was primarily conducted through social media, but the study was also advertised in newspapers, and posters were put up in public places in various cities in Sweden. Interested people were referred to the study's website (https://www.postcoronacope.se) for information about the study, registration, and inclusion and exclusion criteria. Registration consisted of filling out online informed consent and the pre-treatment measurement, involving demographic questions and the following questionnaires: Beck Depression Inventory-II (BDI-II), Generalized Anxiety Disorder-7 (GAD-7), Perceived Stress Scale-14 (PSS-14), Insomnia Severity Index (ISI), Short Health Anxiety Inventory-14 (SHAI-14), Alcohol Use Disorders Identification Test (AUDIT), Karolinska Exhaustion Disorder Scale-9 (KEDS-9), Impact of Event Scale-6 (IES-6), University of California Los Angeles Loneliness Scale–Revised (UCLA-LS-R), Brunnsviken Brief Quality of Life Scale (BBQ), The Cognitive Failures Questionnaire (CFQ), and InCharge Financial Distress/Financial Well-Being Scale (IFDFW). In addition, questions about earlier experiences with psychological treatment and a knowledge test about CBT, constructed by the research team, were also included. See below for more details about the pre-treatment measurement and questionnaires.

After registration, individuals were contacted by telephone for a semi-structured clinical interview (see Supplementary Material), asking them in-depth questions about their reasons for participating in the study. The interview aimed to investigate whether the treatment could be helpful to them and, if so, which modules that could be relevant depending on each individual's unique situation. All cases were thoroughly discussed by the research team based on the inclusion and exclusion criteria, see below. In case of exclusion, the individual was contacted by the research team via email, providing reasons for excluding the person. If needed, the excluded individual was referred to other healthcare providers. If the reason for exclusion was based on too severe mental illness, self-harm, or suicidal risk, the individual was contacted by phone.

#### 2.2.2 Inclusion and exclusion criteria

To be included in the study, individuals had to (a) experience psychological symptoms related to the COVID-19 pandemic or its consequences in its final phase (i.e., when the study was conducted), (b) be at least 18 years old, (c) able to read, write and speak Swedish, and (d) have access to a computer or other device with internet connection. Individuals were excluded from the trial if they (a) had mental or somatic problems that would substantially complicate participation or make participation impossible, (b) ongoing addiction, (c) acute suicidality, (d) currently receiving other psychological treatment, or (e) had changed the dose of psychotropic medication or have planned changes during the treatment weeks. Thus, post-COVID syndrome, or any other somatic or psychiatric diagnosis was neither an inclusion nor an exclusion criterion. Inclusion and exclusion criteria were assessed using information from the pre-treatment screening measures and the semi-structured clinical interview.

#### 2.2.3 Participants

**Figure 1:**
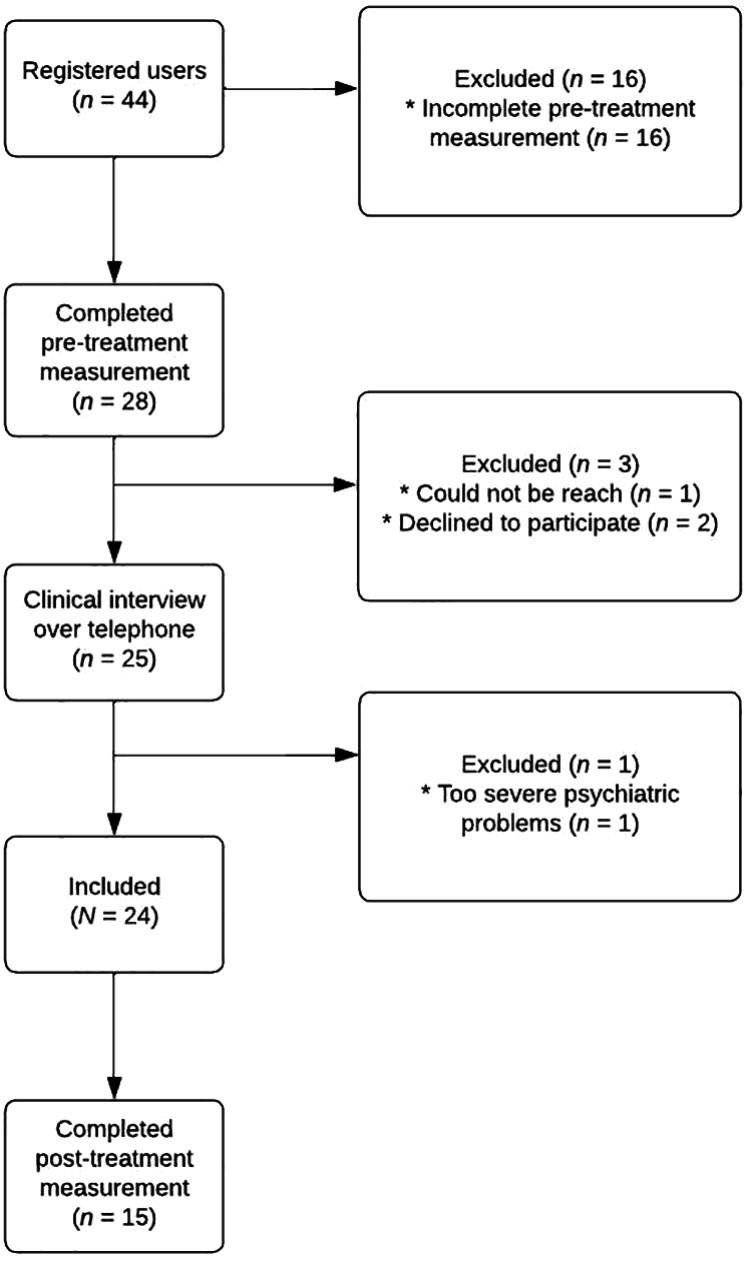
Flowchart of participants in the study.

A flowchart of the recruitment process, including the number participants that completed post-treatment measurement, is presented in Figure 1. In total, 44 individuals gave informed consent and thus expressed interest in participating in the study. A total of 16 (36.4%) of them fulfilled some of the questionnaires required in the pre-treatment measurement, but did not complete it and, thus, did not officially apply for the study. Out of the 28 individuals who fulfilled the pre-treatment screening, one (3.6%) could not be reached by telephone or e-mail, and two (7.1%) declined further participation in the study. Thus, 25 people were interviewed and subsequently assessed for inclusion. After the telephone interview and discussion within the research team, one individual was excluded due to the severity of their psychiatric condition, based on the self-reported questionnaires included in the pre-treatment measurement and the clinical interview (for the interview questions, see Supplementary Material). Based on the information provided, the intervention was deemed not to address the individual's primary concerns. Inclusion and exclusion assessments were made collaboratively by the research team, although the final decision was made by the lead project researcher (GA). Consequently, in total, 24 participants were included in the study.

During the study, there were no physical visits. Thus, it was not possible to assess with certainty whether participants met the criteria for post-COVID syndrome or not. However, 11 (45.8%) participants reported having received the diagnosis, and 5 (20.8%) others reported symptoms that likely could be attributed to a SARS-CoV-2 infection and thereby likely could be categorized as symptoms of post-COVID syndrome. Criteria for assessing “probable post-COVID syndrome” were; (a) typical symptoms of post-COVID syndrome, (b) typical time association with onset of the post-COVID symptoms at the same time as the infection, (c) symptoms persisting for more than twelve weeks after infection onset, and (d) functional impairment due to these symptoms. A physician assessed if these criteria were met for every participant, and this categorization was thereafter confirmed by a specialist in rehabilitation medicine. From this evaluation, it was assessed that 16 (66.7%) out of the 24 participants probably met the diagnosis criteria for post-COVID syndrome.

### 2.3 Data collection

#### 2.3.1 Measures

All questionnaires were administered both at pre- and post-treatment. However, questions about earlier experiences of psychological treatment and other background factors were only used in the pre-treatment measurement. These questions, such as “Do you have any previous experience of psychological treatment?” or “Were there any specific techniques or strategies used?” (for all questions, see Supplementary Material) were included for exploratory purposes and were thus not involved in any statistical analyses. The pre-treatment measurement was completed by participants upon registering for the study. Approximately three weeks elapsed from the study's opening until participants were invited to the treatment platform and the intervention subsequently commenced. The maximum interval between a participant's completion of the pre-treatment measurement and the initiation of treatment was approximately three weeks. The post-treatment measurement was distributed in mid-April 2023, coinciding with participants receiving their final weekly feedback from their therapists. Participants were given a two-month window to complete the post-treatment measurement, with a final deadline at the end of June 2023.

#### 2.3.2 Primary outcomes

##### 2.3.2.1 Beck depression inventory-II

To measure depressive symptoms, the BDI-II was used. The questionnaire consists of 21 items rated on a four-point Likert scale (47). Thus, the total score ranges between 0 and 63, and a higher score indicates a higher level of depressive symptoms. The score is interpreted as minimal (0–13), mild (14–19), moderate (20–28) or severe (29–63) depression. The internal consistency is excellent (*α* = .92), and the test–retest reliability is good (*r* = .93) (47). In the present sample, the internal consistency at baseline was good (*α* = .88).

##### 2.3.2.2 Generalized anxiety disorder-7

The GAD-7 was used to evaluate symptoms of anxiety and worry. The respondent first answers seven items on a four-point Likert scale from 0 (Not at all) to 3 (Almost every day) and then answers a question about whether the symptoms estimated on the first seven items affects the ability to work, household chores, or relationships (48). The maximum total score for the first seven questions is 21, with higher scores mirroring higher levels of symptoms. Cut-off scores for mild, moderate, and severe symptoms are 5, 10, and 15. The measure has been shown to have both excellent internal consistency (*α* = .92) and good test–retest reliability (*r* = .83) (48). The internal consistency was good in the current sample (*α* = .88).

#### 2.3.3 Secondary outcomes

##### 2.3.3.1 Perceived stress scale-14

Stress symptoms were measured using the PSS-14, a questionnaire of 14 items rated on a five-point Likert scale, from 0 (Never) to 4 (Very often). Consequently, the total score ranges between 0 and 56, and higher scores reflect higher levels of stress symptoms (49). Good psychometric properties have been shown with Cronbach's *α* ranging from .75 to .89, even though the test–retest reliability has been seldom evaluated (50). The internal consistency within this study was good (*α* = .89).

##### 2.3.3.2 Insomnia severity index

The ISI is a self-report questionnaire aiming to measure insomnia symptoms with its seven items (51). With a five-point Likert scale, from 0 (Not at all) to 4 (Very much), the total score ranges between 0 and 28. The scores are interpreted as; probably no significant sleep problems (0–7), some sleep problems (8–14), moderate insomnia (15–21), and severe insomnia (22–28). According to Bastien et al. (51), the ISI has acceptable internal consistency (*α* = .74) and good test–retest reliability (*r* = .78). In the present sample, the internal consistency was good (*α* = .89).

##### 2.3.3.3 Short health anxiety inventory-14

To measure symptoms of health anxiety, the questionnaire SHAI-14 was used. It consists of 14 items, each scored on a four-point Likert scale (52). A higher score, with a maximum of 42, reflects higher levels of health anxiety symptoms. A score less than or equal to 14 is interpreted as a low probability of health anxiety, while a score equal to or more than 18 is interpreted as a high probability of health anxiety. The SHAI-14 has been shown to have good internal consistency (*α* = .89) and test–retest reliability (*r* = .87) (52). The internal consistency in the current sample was excellent at baseline (*α* = .91).

##### 2.3.3.4 Karolinska exhaustion disorder scale-9

With the KEDS-9, symptoms of fatigue and exhaustion are aimed to be measured. It consists of nine items, including questions about the ability to concentrate, memory, and recovery (53). The items are scored on a seven-point Likert scale, from 0 to 6, which gives a total score range between 0 and 54. A higher score indicates higher levels of exhaustion symptoms. The cut-off score for stress-related exhaustion problems is 19, which has been shown to be accompanied by high sensitivity and specificity (each above 95%). Research on test–retest reliability is limited, but the KEDS-9 has been shown to have acceptable internal consistency (*α* = .74 and higher) (53). At baseline, in the present sample, the internal consistency was good (*α* = .87).

##### 2.3.3.5 Impact of event scale-6

To screen for post-traumatic stress symptoms, IES-6 was used. It is an abbreviated version of the Impact of Event Scale–Reversed (IES-R), and it is highly correlated with the IES-R across samples (54). The IES-6 consists of six statements, and the responder is asked to estimate, on a five-point Likert scale, how disturbing a life event has been during the last seven days, from 0 (Not at all) to 4 (Extremely). The total score range is between 0 and 24. When investigated, the IES-6 was shown to have good internal consistency (*α* = .80) (54). The internal consistency at baseline within this study was excellent (*α* = .91).

##### 2.3.3.6 University of California Los Angeles Loneliness scale–revised

The UCLA-LS-R was used to measure experiences of loneliness. It contains 20 items, and the responder rates the items on a four-point Likert scale, from 1 (Never) to 4 (Always; Russell et al., 1980). The total score range is between 20 and 80, and a higher score indicates experiences of loneliness to a larger extent. The measure has excellent internal consistency (*α* = .94) (55). Also, in the present sample, the UCLA-LS-R had excellent internal consistency (*α* = .91),

##### 2.3.3.7 Brunnsviken brief quality of life scale

Experienced quality of life was measured by using the BBQ. The questionnaire involves 12 items that cover six areas of life, assessing how important and satisfying the responder experiences them (56). The items are rated on a five-point Likert scale, from 0 (I do not agree at all) to 4 (I totally agree). The scores of pairs of items are multiplied by each other, and then the sum of the factors constitute the total score. The total score ranges between 0 and 96. A higher score reflects a higher experienced quality of life, and the cut-off 52 has been used to differentiate between a clinical and a non-clinical group. The psychometric properties have shown to be good, with a high test–retest reliability (ICC = .86) and acceptable internal consistency (*α* = .76) (56). The internal consistency in the present sample was good (*α* = .84).

##### 2.3.3.8 The cognitive failures questionnaire

Consisting of 25 questions, the self-reported questionnaire CFQ means to measure the respondent's experience of their memory function, perception, and motor function (57). The responder rates how often the questions apply to oneself on a five-point Likert scale, from 0 (Never) to 4 (Very often), during the last six months. The total score range is between 0 and 100, and a higher score indicates that problems with cognitive failure are experienced to a greater extent. Scores ≥43 are interpreted as high (57). Test–retest reliability has shown to be acceptable (*r* = .71) (58) and the internal consistency to be excellent (*α* = .94) (59). In the present study, the internal consistency was excellent (*α* = .92).

##### 2.3.3.9 InCharge financial distress/financial well-being scale

To measure the level of financial stress and well-being, the IFDFW was used. The questionnaire consists of eight items, and the respondents rate them on a scale between 1 and 10 (60). The total score is calculated by adding all scores on each item, divided by eight (i.e., number of items). Thus, the total score range is between 1 and 8, where a higher score reflects higher financial well-being (or lower financial stress). Total scores of 1–4 are interpreted as high financial distress/low financial well-being, and 7–10 are interpreted as low financial distress/high financial well-being. Prawitz et al. (60) demonstrated that the IFDFW has excellent internal consistency (*α* = .96). In the present sample, the internal consistency was high (*α* = .93).

#### 2.3.4 Other measures

##### 2.3.4.1 Alcohol use disorders identification test

The AUDIT involves ten items about the respondent's alcohol use, harmful effects of consuming alcohol, and dependency symptoms (61). Responses to each item are scored between 0 and 4, which gives a total score range from 0 to 40 (61). The measure has been evaluated extensively and has favorable validity and reliability (62). The AUDIT has good internal consistency (*α* = .82) (63), and high test–retest reliability (*r* = .97) (64). In the present study, the internal consistency for AUDIT was *α* = .84. Primarily, the AUDIT was used to screen for alcohol use in this study and, therefore, was not analyzed as a separate outcome measure.

##### 2.3.4.2 Knowledge test

A knowledge test about CBT principles, based on a knowledge test developed by Berg et al. (65), was also used. The knowledge test can be found in Supplementary Material. The knowledge test included items such as “According to CBT, what happens if you avoid a harmless situation that triggers anxiety?”. The test consisted of 16 items, and since the original was developed for adolescents, it was adopted for an adult population. Every question had three alternative responses, including one correct and two incorrect answers. A correct answer gets one point, and an incorrect answer gets zero points. The test investigates whether the respondent can identify the correct CBT principle in relation to different situations and problems. In addition to answering the specific questions in the knowledge test, respondents also indicate their confidence in their answers on a three-point Likert scale, ranging from 1 (“I am guessing”) to 3 (“I am entirely sure”). A total score based on the number of correct answers (raw score) and a weighted score was calculated, with the raw score weighted by the confidence ratings. Thus, the raw score was based solely on the total number of correct answers on the knowledge test. The weighted score included the level of certainty in the calculation, in addition to the raw score. If a respondent answered correctly and was entirely sure, the score obtained was +1. If a respondent answered correctly but was uncertain or guessing, the score obtained was 0. The score was also 0 if the respondent answered incorrectly and was uncertain or guessing. If the respondent answered incorrectly and was entirely sure, the score obtained was −1 (65). Thus, the total for the raw score ranged from 0 to 16, and for the weighted score from −16 to 32. The Cronbach's *α*, calculated on the raw score for the knowledge test, was shown to be .82 in the present study.

##### 2.3.4.2 Patient health questionnaire-9

The PHQ-9 was also used in this trial, but only to monitor the participants' well-being during the treatment. The PHQ-9 was administered once a week. Thus, it was not included in the pre- and post-treatment measurements. PHQ-9 is a 9-item self-reported questionnaire aiming to measure depressive symptoms and includes a question about thoughts of death or self-harm (66). Response options are on a four-point Likert scale, from 0 (Not at all) to 3 (Nearly every day), and higher scores indicate higher symptom severity (66).

### 2.4 Platform and intervention process

Questionnaires, the treatment, including the distribution of the modules, and all communication between therapists and participants were executed through a secure online platform (67). To access the platform, both the research team, including the therapists, and the participants were required to use their individual user name (for the participants, it was an auto-generated study code, e.g., 1234abcd), password, and a six-letter code sent via SMS to the telephone number associated to the specific login. Once participants logged into the treatment platform, they could access two main features: treatment modules and a messaging inbox. Within the treatment modules section, participants could view the modules to which their therapist had granted access. Each module began with an introductory section welcoming the participant and providing an overview of the module's content. In the messaging inbox, participants could find messages from their therapist as well as the messages they themselves had sent to the therapist, if any were sent.

The treatment was an individually tailored ICBT, where participants received eight modules during eight weeks. The modules can be likened to individual chapters in a book, which do not necessarily need to be read sequentially from beginning to end. Instead, selected chapters may be utilized based on the individual's needs. The modules included psychoeducational texts, models, and content-related exercises. The idea was for the participants to work with one module each week. All participants received the same first module, Introduction, and the same last module, Conclusion and maintenance plan. The Introduction module provides psychoeducation on the mental health impact of the COVID-19 pandemic and CBT. Participants are encouraged to identify life domains that are important to them and to formulate their treatment goals. In the Conclusion and maintenance plan module, the importance of continuing to apply strategies from the treatment that was experienced helpful is emphasized. The participant is guided through a summary of the modules they have completed. Additionally, participants get to create an action plan in case of symptom recurrence, along with a reflection on the treatment goals. The remaining six modules were selected based on the participant's specific problems and situation. This decision was made by the research team, based on pre-treatment measurements and clinical telephone interviews. However, the participants also had the opportunity to wish which modules they wanted to include in their treatment during their work in the first module, Introduction. Thus, modules were chosen for each participant based on the research team's assessment and the individual participant's preferences.

Except for the modules Introduction and Conclusion and maintenance plan, there was a pool of 18 modules to select from. The modules covered the following topics: Behavioral activation, Cognitive restructuring, Acceptance, Emotion regulation, Anxiety and exposure, Anxiety and worry, Social anxiety, Panic, Sleep, Perfectionism, Stress management, Relaxation, Problem-solving, Difficult memories, Focus and concentration, Manage financial stress, Loneliness, Healthy self-assertion. The modules are described further in Supplementary Material. In summary, the modules comprise psychoeducation and standard CBT strategies, with a focus on depressive symptoms, exposure to anxiety and other distressing emotions, mindfulness, problem-solving both individually and in relation to various specific problems, as well as specific themes such as perfectionism and loneliness. Most of the modules were taken from earlier ICBT treatments and studies, mainly from Aminoff et al. (42), and was adopted to the prevailing circumstances in Swedish society during the final phase of the COVID-19 pandemic (at the beginning of the year 2023). For instance, at this time, the pandemic had been ongoing for just over three years, with fluctuations in infection rates, potentially contributing to a sense of uncertainty and hopelessness among the population. Consequently, the Introduction module, as well as the introduction to several other modules, focused on how a prolonged pandemic could affect one's mental health, and specifically how the COVID-19 pandemic has been shown to impact the well-being. Unlike recent ICBT studies during the pandemic conducted by our research team (40, 42), certain exercises, such as exposure, could once again involve physical interactions to some extent. Thus, the modules were partially readjusted to a post-pandemic context, but also to target how an individual might find it challenging to reintegrate, such as reaching out to a friend they have not contacted in a long time. Additionally, four new modules were developed to fit the potential needs of the targeted population; Focus and concentration, Manage financial stress, Loneliness, and Healthy self-assertion.

Participants received weekly support from a therapist based on the exercises connected to the module for each specific week. The support by the therapists and contact between them and the participants were via asynchronous text messages on the treatment platform. In the weekly feedback, therapists provided encouragement regarding the participant's work with the current module, offered suggestions on how engagement with specific exercises could be further developed, and inquired whether the participant needed any assistance to continue working with the treatment. After reading and responding to what the participant had written in a module, the therapist distributed the next module to the participant. During the treatment period, the participants had the opportunity to send messages to their therapist through the treatment platform and then receive answers within 24 h on weekdays. To monitor the participants' psychological well-being during the treatment, PHQ-9 was administered once a week. If a participant rated their well-being as dramatically worsened, the routine was to call the participant for an assessment and gather information for potential actions, such as assisting the individual in seeking care at another facility. The same procedure was carried out if the participant estimated the highest score on the question about having thoughts of death and self-harm included in the PHQ-9.

#### 2.4.1 Therapists

Participants received weekly feedback from final-year students (*n* = 4) studying their last semester in the clinical psychologist program at Linköping University. All students had experience of at least 18 months of theoretical and practical work based on CBT. The students received weekly group supervision from a licensed psychologist throughout both the assessment phase and the treatment period. The therapists were responsible for providing feedback on the module exercises and responding to any questions related to treatment content. All communication occurred asynchronously via text on the treatment platform. The feedback primarily focused on communicative aspects identified by Paxling et al. (68), such as task reinforcement (giving verbal reinforcement for strategies and behaviors aligned with the module's rationale) and empathic expression (showing empathy towards experienced difficulties and struggles).

### 2.5 Statistical analyses

All statistical analyses were performed in IBM SPSS Statistics Version 29. Significance testing was two-tailed, and results were interpreted as significant at *p* < .05. Complete Case Analysis (CCA) was conducted, whereby only participants who completed the post-treatment measurement were included in the analysis of treatment outcomes. This was in line with an Intention-To-Treat approach. Thus, all participants fulfilling the post-treatment measurement were included in the outcome analysis, regardless of how many modules they had worked with. Dropout analyses were performed with independent samples *t*-test for continuous variables and *χ*^2^-test for categorical variables.

Assumptions for the general linear model were tested, i.e., normality, linearity, and homogeneity of variance. Treatment outcome analysis was performed using the Wilcoxon signed-rank test to evaluate whether estimated symptoms had changed from pre- to post-treatment measurement. Effect size, the correlation coefficient *r*, was calculated by dividing the *z*-score from the Wilcoxon test with the square root of the total number of observations (69). Guidelines for interpretation are the same as for Pearson's correlation coefficient (70), namely *r* < 0.30 is interpreted as small, *r* between 0.30 and 0.49 as moderate, and *r* ≥ 0.50 as large. Paired samples *t*-test was also used for robustness test. With the *t*-test, the standardized mean difference (Cohen's *d*) was used as effect size, following the guidelines for interpretation by Cohen (71) where *d* = 0.20 is assessed as small, *d* = 0.50 is as moderate and *d* = 0.80 as large effect.

## 3 Results

### 3.1 Participants

Demographics for the 24 participants who were included in the study are displayed in Table 1. Their age ranged from 20 to 67 years, with a mean age of 40.83 (*SD* = 15.9). Sixteen (66.7%) of them were females, 7 (29.2%) were men, and 1 (4.2%) identified themselves as “other”. Sixteen (66.7%) had previous experience of psychological treatment and 10 (41.7%) used psychopharmaceutic medication. Twenty (83.3%) had tested positive for SARS-CoV-2 infection at some point and all 24 (100%) were vaccinated for COVID-19.

**Table 1:** Demographics (*N* = 24).

Characteristics	*M* (*SD*) or *n* (%)
Age in years, *M* (*SD*)	40.83 (15.9)
Age in years, min–max	20–67
Gender	
Females, *n* (%)	16 (66.7)
Men, *n* (%)	7 (29.2)
Other, *n* (%)	1 (4.2)
Education level, *n* (%)	
Secondary school	3 (12.5)
Vocational training	7 (29.2)
College/University (ongoing)	4 (16.7)
College/University (finished)	9 (37.5)
Other	1 (4.2)
Occupational status, *n* (%)	
Student	2 (8.3)
Working	10 (41.7)
Retired	3 (12.5)
Registered sick leave	5 (20.8)
Other	4 (16.7)
Use of psychopharmaceutic medication, *n* (%)	
No	14 (58.3)
Yes, previously	0 (0)
Yes, ongoing	10 (41.7)
Experience of psychological treatment	
No	5 (20.8)
Yes, previously	16 (66.7)
Yes, ongoing	3 (12.5)
Somatic problems	
No	10 (41.7)
Yes	14 (58.3)
Tested positive for SARS-CoV-2 virus	
No	2 (8.3)
Yes	20 (83.3)
Uncertain	2 (8.3)
Vaccinated for COVID-19	
No	0 (0)
Yes	24 (100)

### 3.2 Attrition, treatment adherence, and therapist time

Out of the 24 participants included, 3 (12.5%) explicitly wished to discontinue during the treatment weeks. On average, the participants completed 3.96 (*SD* = 3.38) modules. A completed module was defined as a module in which the participant, through written reflections in the exercises included in the specific module or through messages to the therapist, demonstrated an understanding of the module's core content. A total of 12 (50%) participants completed over four modules, and 5 (20.8%) participants completed all eight assigned modules. No significant correlation was found between number of completed modules and change in estimated symptoms from pre- to post-treatment measurement, except for experienced quality of life, measured with the BBQ. Increases in experienced quality of life were positively correlated with a higher number of completed modules (*r* = .61, *p* = .015). The number of times participants logged into the ICBT platform ranged from 0 to 46, with a mean of 19.67 (*SD* = 14.41).

The therapists' average time per participant was 119.63 (*SD* = 85.26). Consequently, the average weekly therapist time per participant was 14.95 (*SD* = 12.86) minutes. Correlations between therapist time and change in self-rated symptoms from pre- to post-treatment measurement were significant for stress symptoms, measured with the PSS-14 (*r* = .58, *p* = .024), and for exhaustion symptoms, measured with the KEDS-9 (*r* = .64, *p* = .011). Thus, more therapist time was associated with a greater reduction in self-rated symptoms of stress and exhaustion.

### 3.3 Missing data

As shown in the flowchart (see Figure 1), 15 (62.5%) out of the 24 participants completed post-treatment measurements. Thus, the study had a drop-out rate of 37.5% (9 participants). No significant differences in pre-treatment measures (all *p*'s > .141) were identified between participants who did (completers) and participants who did not (non-completers) complete the post-treatment measurement. Using a *χ*^2^-test, a significant difference in education level between the completers and non-completers (*p* = .007) was found, see Table 2. More completers seemed to have an education level at college/university level than the non-completers. The completers and non-completers did not significantly differ regarding age, gender, or occupational status.

**Table 2:** The educational level of the participants, divided by whether the participants completed the post-treatment measurement or not.

Education level	Participants (*n*) that did not complete the post-treatment measurement	Participants (*n*) that did complete the post-treatment measurement
Secondary school	3	0
Vocational training	4	3
College/University (ongoing)	1	3
College/University (finished)	0	9
Other	1	0

### 3.4 Treatment outcome

Table 3 shows descriptive statistics for the sample in total (when using CCA) for pre- and post-treatment measurement. Ten (66.7%) out of 15 participants who completed the post-treatment measurement were assessed as likely meeting the diagnosis criteria for post-COVID syndrome. The descriptive statistics divided by whether the participants were likely to meet the diagnosis criteria for post-COVID syndrome or not are displayed in Table 4.

**Table 3:** Descriptive statistics pre- and post-treatment.

Measure	Pre-treatment (*n* = 15)			Post-treatment (*n* = 15)		
	*M*	*SD*	*Mdn*	*M*	*SD*	*Mdn*
BDI-II	23.27	10.07	22.00	14.07	9.16	13.00
GAD-7	8.13	6.71	5.00	5.47	5.28	4.00
PSS-14	32.00	9.60	34.00	27.87	9.22	27.00
ISI	15.40	7.52	15.00	12.67	8.53	16.00
SHAI-14	20.20	9.52	22.00	15.53	9.07	16.00
KEDS-9	30.07	10.31	31.00	26.13	12.02	24.00
IES-6	9.67	7.13	10.00	5.33	6.21	3.00
UCLA-LS-R	46.73	8.84	46.00	42.27	7.81	42.00
BBQ	37.73	19.99	41.00	43.47	22.21	40.00
CFQ	49.67	15.75	48.00	46.13	20.30	38.00
IFDFW	5.66	2.26	5.63	6.07	2.43	6.00
Knowledge test						
Raw score	13.33	2.19	14.00	14.40	2.01	16.00
Weighted score	17.53	6.05	15.00	21.87	6.83	22.00

M, mean; SD, standard deviation; Mdn, median; BDI-II, Beck Depression Inventory-II (depressive symptoms); GAD-7, Generalized Anxiety Disorder-7 (anxiety and worry symptoms); PSS-14, Perceived Stress Scale-14 (stress symptoms); ISI, Insomnia Severity Index (insomnia symptoms); SHAI-14, Short Health Inventory-14 (health anxiety symptoms); KEDS-9, Karolinska Exhaustion Disorder Scale-9 (fatigue and exhaustion symptoms); IES-6, Impact of Event Scale-6 (post-traumatic stress symptoms); UCLA-LS-R, University of California Los Angeles Loneliness Scale – Revised (experiences of loneliness); BBQ, Brunnsviken Brief Quality of Life Scale (experienced quality of life); CFQ, The Cognitive Failures Questionnaire (experience of memory function, perception, and motor function); IFDFW, InCharge Financial Distress/Financial Well-Being Scale (financial stress and well-being).

**Table 4:** Descriptive statistics for pre- and post-treatment measurement, categorized by whether the participants probably fulfilled the diagnosis criteria for post-COVID syndrome or not.

Measure	Probably post-COVID (*n* = 10)		Probably not post-COVID (*n* = 5)	
	Pre-treatment *M* (*SD*)	Post-treatment *M* (*SD*)	Pre-treatment *M* (*SD*)	Post-treatment *M* (*SD*)
BDI-II	23.30 (11.05)	15.20 (10.15)	23.20 (8.89)	11.80 (7.23)
GAD-7	7.10 (6.89)	4.9 (5.26)	10.20 (6.54)	6.60 (5.73)
PSS-14	31.30 (10.52)	26.90 (10.19)	33.40 (8.36)	29.80 (7.53)
ISI	15.50 (7.91)	12.20 (9.11)	15.20 (7.56)	13.60 (8.14)
SHAI-14	20.90 (6.66)	17.00 (8.59)	18.80 (14.62)	12.60 (10.29)
KEDS-9	31.90 (11.11)	28.60 (13.04)	26.40 (8.30)	21.20 (8.79)
IES-6	9.60 (7.17)	5.20 (6.53)	9.80 (7.89)	5.60 (6.23)
UCLA-LS-R	45.50 (8.20)	40.90 (7.49)	49.20 (10.52)	45.00 (8.54)
BBQ	40.40 (21.03)	45.20 (24.33)	32.40 (18.72)	40.00 (19.27)
CFQ	52.40 (17.92)	51.30 (22.87)	44.20 (9.47)	35.80 (8.04)
IFDFW	6.17 (2.41)	6.03 (2.70)	4.99 (2.44)	6.15 (2.05)
Knowledge test				
Raw score	14.10 (1.60)	14.60 (1.78)	11.80 (14.80)	14.00 (2.83)
Weighted score	18.90 (6.90)	22 (6.46)	2.59 (2.59)	21.60 (8.33)

M, mean; SD, standard deviation; BDI-II, Beck Depression Inventory-II; GAD-7, Generalized Anxiety Disorder-7; PSS-14, Perceived Stress Scale-14; ISI, Insomnia Severity Index; SHAI-14, Short Health Inventory-14; KEDS-9, Karolinska Exhaustion Disorder Scale-9; IES-6, Impact of Event Scale-6; UCLA-LS-R, University of California Los Angeles Loneliness Scale–Revised; BBQ, Brunnsviken Brief Quality of Life Scale; CFQ, The Cognitive Failures Questionnaire; IFDFW, InCharge Financial Distress/Financial Well-Being Scale.

Assumptions for the general linear model (including paired samples *t*-test) were investigated. Differences between pre-and post-treatment scores were assessed to be approximately normally distributed for all measures, using the Kolmogorov–Smirnov test and histogram. This is except for the GAD-7, where the Kolmogorov–Smirnov test was significant (*p* = .034), and for the ISI, where the Kolmogorov–Smirnov test was significant too (*p* < .001). Outliers were detected for the following outcome measures: the BDI-II, the GAD-7, the ISI, the SHAI-14, the UCLA-LS-R, the CFQ, and the IFDFW.

Considering the present outliers, we primarily conducted a non-parametric test, the Wilcoxon signed-rank test, but also calculated the treatment outcomes with parametric tests, with paired samples *t*-test, to compare the results as a robustness tests.

#### 3.4.1 Wilcoxon signed-rank test

To investigate treatment effects, the Wilcoxon signed-rank test was performed, and effect sizes (*r*) were calculated by dividing the standardized test statistic (*z*) by the square root of the number of observations (Rosenthal, 1991). The number of observations is 30 (pre- and post-treatment symptom estimates by 15 participants), and the square root of 30 is roughly 5.477.

For the BDI-II, the post-treatment measure estimate of depression symptoms was significantly lower (*Mdn* = 13.00) than the pre-treatment measure estimate (*Mdn* = 22.00), *T* = 4.50, *p* = .003. This with a large effect size, *r* = 0.53. For the GAD-7, the other primary outcome measure, the post-treatment measure estimate of anxiety symptoms was also significantly lower (*Mdn* = 4.00) than the pre-treatment measure estimate (*Mdn* = 5.00), *T* = 7.00, *p* = .020. This with a moderate effect size, *r* = 0.43.

Regarding secondary outcome measures, the SHAI-14 estimating health anxiety significantly differed between post-treatment (*Mdn* = 16.00) and pre-treatment (*Mdn* = 22.00) measurement, *T* = 11.50, *p* = .010. The post-treatment measurement was lower than the pre-treatment measurement, with a moderate effect size, *r* = 0.47. Similar effects were shown for the KEDS, measuring symptoms of fatigue and exhaustion, between post-treatment (*Mdn* = 24.00) and pre-treatment (*Mdn* = 31.00) measurement, *T* = 24.00, *p* = .041, *r* = 0.37. This was shown for the IES-6 as well, measuring post-traumatic stress symptoms, showing significant lower estimates at post-treatment (*Mdn* = 3.00) than pre-treatment (*Mdn* = 10.00) measure, *T* = 17.00, *p* = .025, *r* = 0.41. A moderate effect (*r* = 0.40) was shown for the UCLA-LS-R, measuring experiences of loneliness, where post-treatment (*Mdn* = 42.00) was lower than pre-treatment (*Mdn* = 46.00) estimate, *T* = 21.00, *p* = .027. Knowledge test, with raw scores, showed to significantly differ with higher scores post-treatment (*Mdn* = 16.00) than pre-treatment (*Mdn* = 14.00), *T* = 60.00, *p* = .014, with a moderate effect size (*r* = 0.45). A significant moderate difference (*r* = 0.40) was also found for the knowledge test on the weighted total score, *T* = 87.50, *p* = .028, where the post-treatment scores (*Mdn* = 22.00) were higher than pre-treatment scores (*Mdn* = 15.00).

In contrast to other measures assessing psychological symptoms, the post-treatment scores on ISI, measuring insomnia symptoms, were significantly higher (*Mdn* = 16.00) than pre-treatment (*Mdn* = 15.00), *T* = 24.00, *p* = .039, with a moderate effect size (*r* = 0.38). The results, thus, indicate increasing symptoms of insomnia post-treatment in comparison to pre-treatment.

No significant differences between pre- and post-treatment measurement were found for the PSS-14, which measures stress symptoms (*p* = .147, *r* = 0.26), the BBQ, which measures the experienced quality of life (*p* = .346, *r* = 0.17), the CFQ, which measures experiences of cognitive functioning (*p* = .118, *r* = 0.29), and the IFDFW, which measures financial stress and well-being (*p* = .977, *r* = 0.01).

#### 3.4.2 Paired samples *t*-test

As a robustness check, we also performed paired samples *t*-test, using bias-corrected accelerated (BCa) for estimating confidence intervals. In summary, the same results were shown by the *t*-tests as by the Wilcoxon signed-rank tests, with two exceptions.

One exception was for the ISI, which with the Wilcoxon signed-rank test showed to significantly increase. With the *t*-test, no significant difference between pre- and post-treatment measurement was found for the ISI (*p* = .077, *d* = 0.49). The other exception was for the KEDS, which with the Wilcoxon signed-rank test showed to decrease. With the *t*-test, no significant difference between pre- and post-treatment measurement was found for the KEDS (*p* = .057, *d* = 0.54).

## 4 Discussion

This study aimed to investigate the feasibility of an eight-week individually tailored ICBT with weekly guidance during the final phase of the COVID-19 pandemic. It was planned to be an RCT, comparing a treatment group with a waitlist control condition. However, we decided to switch to a within-group design due to recruitment issues and the low number of included participants. Out of the 25 individuals who went through the pre-treatment screening, 24 were included, and 15 completed the post-treatment measurement. Three participants explicitly expressed their desire to withdraw from the study during the treatment period. Out of eight assigned modules, the participants completed an average of 3.96 modules. Comparing pre-treatment and post-treatment estimates of various psychological symptoms using self-assessment questionnaires, results revealed significant decreases in depression and anxiety. Significant decreases were also found for some secondary outcomes (symptoms of health anxiety, fatigue and exhaustion, post-traumatic stress, loneliness) but not for others (stress, quality of life, experiences of cognitive functioning, financial stress and well-being). Knowledge about CBT principles was found to increase, as well as insomnia symptoms (even if the robustness test did not show increases in insomnia). Overall, the findings indicate that ICBT is feasible as a treatment for psychological symptoms during a pandemic, and more specifically, was feasible during the end of the COVID-19 pandemic. However, some adjustments to the treatment would be beneficial and necessary to improve and investigate further, to ensure its effectiveness.

This study was conducted to assess the feasibility of the ICBT in the late COVID-19 pandemic. The outcome analyses showed that symptoms of depression, anxiety, post-traumatic stress, healthy anxiety, loneliness, fatigue, and exhaustion, measured with the BDI-II, the GAD-7, the IES-6, the SHAI-14, the UCLA-LS-R, and the KEDS-9 respectively, had decreased from pre- to post-treatment measurement with medium to large effect sizes. Scores on the knowledge test about CBT, both raw and weighted, increased. Robustness tests showed the same results, except for the KEDS, which was not statistically significant. Thus, effects on primary and some of the secondary outcome measures were shown. The results are in line with earlier studies of ICBT conducted before (72) and during the COVID-19 pandemic (31), which show symptom reduction associated with ICBT, and an increased knowledge about CBT (65). The modules in the treatment included typical CBT strategies, but the content was adapted to fit problems related to the final phase of the COVID-19 pandemic. Regarding the primary outcome measures, symptoms of depression and anxiety, the results are consistent with previous findings from studies evaluating most of the treatment modules used in this study (40, 42). These findings further strengthen the evidence that ICBT, including weekly therapist support, can be beneficial not only at the onset (40) or during the middle phase of the COVID-19 pandemic (42), but also in its final phase. The results also suggest that the individually tailored approach, where participants receive treatment plans consisting of different modules with varying focus, combined with weekly support from a therapist, is feasible in a pandemic context. Module selection was based on a combination of the research team's clinical judgment and the participant's own preferences. These approaches have previously been tested both independently and comparatively, demonstrating effectiveness in reducing depressive symptoms, as well as symptoms of anxiety and quality of life (27). Furthermore, consistent with previous studies (65, 73), an increase in CBT knowledge was identified post-treatment compared to pre-treatment. It is relevant to further investigate CBT knowledge and its development in the context of ICBT, as this treatment format, through its psychoeducational content, may be regarded as a form of patient education and may provide insights into the educational component of the intervention. Overall, the findings indicate that individually tailored ICBT remains a relevant and beneficial intervention even in a global context different from than the one we are accustomed to, such as during a pandemic. The combined approach to module selection, balancing clinical assessment and participant preference, appears to be a viable method for heterogeneous psychological symptoms in times of a pandemic, and potentially other societal transitions.

Except for the effects on fatigue and exhaustion, the results appear to be robust despite the small number of participants and, in that sense, low power. During the COVID-19 pandemic, several previous studies have reported findings regarding transdiagnostic ICBT being effective for depressive and anxiety symptoms (e.g., 35, 38, 74). These transdiagnostic interventions are comparable to the individually tailored ICBT employed in the present study, as the treatment is designed to be applicable to individuals with a wide range of symptoms. In addition to the inclusion of standard CBT strategies in the treatment content, several other factors may contribute to the perceived helpfulness of ICBT. The therapeutic alliance, as in traditional face-to-face therapy, between the participant and the therapist may be important (75), as well as the participant's perceived alliance with the treatment program itself (76). Unfortunately, perceived alliance with the therapist or the ICBT program was not assessed in the present study. Furthermore, when comparing ICBT interventions, it is essential to consider that they often differ in several aspects, including the specific content of interventions (despite being grounded in CBT principles), the presence or absence of therapist support, as well as the overall duration and intensity of the treatment. An additional challenge when comparing ICBT studies conducted during the COVID-19 pandemic is that they were carried out at different phases of the pandemic. There is a considerable variation in the studies conducted, with a range from a brief, self-guided ICBT intervention conducted over three weeks at the beginning of the pandemic (33), to a therapist-guided nine-week intervention implemented during the mid-phase of the pandemic (74). Nevertheless, both approaches demonstrated significant effects on symptoms of anxiety and depression. Thus, despite differences and present challenges for cross-study comparisons, ICBT appears generally to be an effective treatment option for psychological distress during the COVID-19 pandemic (31, 38).

However, it is essential to emphasize that the results in this study were not compared against a control group, which means that other factors, such as the pandemic coming to an end or individual life events, may have impacted the results. Insomnia symptoms, measured with the ISI, did significantly increase during the treatment period, but this was not significant with the robustness test. Systematically, increased insomnia symptoms have, to our knowledge, not been shown during an ICBT, and one of the modules had sleeping problems as a focus, even if not all participants worked with it. It is unclear why the insomnia symptoms increased. No other symptom estimates showed an increase, but if this result reoccurs, it should be further investigated.

Results were non-significant on some of the secondary outcome measures; the BBQ, the PSS-14, the CFQ, and the IFDFW, aiming to measure quality of life, stress symptoms, experience of impairment in memory, perception, and motor function, and financial stress/well-being, respectively. In addition to the risk that this could be due to insufficient power, the treatment did not include modules that focused on all these specific areas, such as experienced cognitive dysfunction. However, there was a module about stress and one on financial stress, but not all participants were assigned these modules. In fact, only two participants completed the module Stress management, and only one completed the module about Managing financial stress. Possibly, a longer treatment period might have been needed for a more noticeable difference in these kinds of symptoms, such as experienced financial stress. Additionally, the treatment could have focused more on somatic issues, such as mental fatigue or pain, which might have had different effects on quality of life, given the number of participants probably having post-COVID syndrome. Nevertheless, there are other examples of ICBT during the COVID-19 pandemic that did not demonstrate significant effects on perceived quality of life among participants without post-COVID syndrome (74). An increase in quality of life between the pre- and post-treatment measurement, measured with the BBQ, was found to correlate significantly with the number of completed modules (*r* = .615). However, determining cause and effect is difficult. As work in the treatment progresses, quality of life may improve, but the ability to work with and complete modules might also partially depend on the experienced quality of life, corresponding to possibly a bidirectional association.

The ICBT investigated was individually tailored, meaning that participants received different modules with different focuses and CBT strategies, depending on their described symptoms. This aligns with the fact that people were affected by the pandemic in different ways (1), as well as with what Treanor et al. (77) concludes in their review about ICBT, that “one size does not fit all”. However, there was a significant difference in education level between those who completed the post-treatment measurement and those who did not, with more completers having an education at a college or university level than the non-completers. Although there could be several reasons for this, a reasonable hypothesis is that the ability and habit of processing text might influence whether a person completes the post-treatment measurement and the treatment in this study, which is primarily delivered via text. While this aligns with previous ICBT studies (78), it may be essential to consider that participants could suffer from cognitive impairments, such as concentration difficulties or mental fatigue, as a result of COVID-19 infection (19, 20). Three participants explicitly stated that they no longer wanted to be part of the study, mainly due to the amount of text in the treatment and because they did not have the energy or time to work through the material. These findings are important for evaluating the feasibility of the ICBT intervention, as well as for identifying which individuals it may, and may not, be suitable for and the underlying reasons.

The mean adherence, in terms of completed modules, was approximately half (3.96 out of 8). This suggests that many participants, given the current format of the modules, do not engage with, or at least do not actively work through, all the material included in the intervention. Although this observation does not differ substantially from previous studies on ICBT conducted during the COVID-19 pandemic (40, 42) or before the pandemic (79), the relatively low adherence may suggest a need for adjustments to the treatment format. In the future, the treatment and the accompanying module texts could be shortened to potentially facilitate the uptake or include both longer and shorter versions of each module, allowing participants to choose a version based on their needs and preferences. One challenge in doing so is determining which content and exercises should be prioritized, as the current treatment material consists of interconnected components, each with an intended purpose. To investigate this, focus groups could be employed, allowing participants to engage with the modules and subsequently provide feedback on which components they found most helpful and which they considered less central. Individual interviews or a survey could also be conducted to investigate participants' experiences regarding which aspects of the treatment were helpful and useful, and which were not.

Sixteen of the 24 participants were assessed as probably having post-COVID syndrome, and the majority of those who completed the post-treatment measurement (ten out of 15) were participants assessed as probably having post-COVID. These were the participants whose data were included in the analysis of treatment outcomes. There are indications that CBT for fatigue in the context of post-COVID may be helpful (80), although the body of research on psychological interventions for post-COVID, both in terms of feasibility and treatment effects, remains limited (81). Even if there is a risk that a text-based treatment, such as ICBT, can be difficult for people with fatigue or other cognitive impairments caused by post-COVID syndrome, another possibility is that text-based modules could for some people be more accessible and usable, since they can read at their own pace and revisit the material without any limitations. This is a contrast to face-to-face treatment, which is primarily verbal, as verbal language is ephemeral. Additionally, the possibility of conducting psychological treatment from home, as enabled by ICBT, could be advantageous for individuals with post-COVID syndrome, or others experiencing somatic symptoms such as pain (82). This positions ICBT as a treatment modality with the potential to reach a wide range of patients (80).

This study's dropout rate was 37.5%, meaning that nine out of 24 participants did not complete the post-treatment measurement. Carlbring et al. (72) noted in their meta-analysis that dropout is expected in CBT and ICBT studies. However, the calculated dropout rate from the meta-analysis was 15.7% (72), which is lower than the rate in this study. Meanwhile, another study of dropout rates by Schmidt et al. (78) estimated the average rate within ICBT for depression to be 32%. Similarly, van Beugen et al. (83) reported a median dropout rate of 29% in ICBT for chronic illness, although the reported range was broad (2%–57%). It is important to acknowledge that these studies were conducted before the outbreak of the COVID-19 pandemic, and that dropout tendencies may differ during and after the pandemic. To the best of our knowledge, dropout rates in ICBT during the final phase of the COVID-19 pandemic have not been specifically investigated and, thus, partly provide the rationale for conducting a feasibility study. Dropout rates during the COVID-19 and other pandemics require further investigation, as well as the reasons for dropping out and the treatment experiences of these participants.

There are several possible reasons for our difficulties in recruiting participants. The study and the recruitment phase were in line with its purpose in the late COVID-19 pandemic, when many were possibly quite tired of the pandemic and were trying to return to some “normal” state, i.e., to the pre-pandemic life and habits. The project's name, PostCoronaCope, could also have been quite unclear and misleading, indicating that the study and treatment were targeted towards post-COVID syndrome, even if no inclusion criteria required that diagnosis or symptoms. One sign of this was the relatively high proportion (66.7%) of individuals with probable post-COVID syndrome included in the study. This, together with the multitude of mental symptoms shown in people with post-COVID syndrome (20), highlights the need for psychological interventions that go beyond efforts aimed at addressing the somatic symptoms caused by COVID-19, even if this was not the primary target population of the present study. Another possible reason for the recruiting difficulties could be that there was less need for treatment for psychological symptoms related to the pandemic or its consequences, even though earlier studies have shown that virus outbreaks can still impact mental health after they end (84–87). Furthermore, the individuals in the present study had to answer multiple online questionnaires included in the pre-treatment measurement. Sixteen individuals began the measurement but did not complete it. At the same time, all questionnaires were administered based on the need to gather thorough information and assess whether the treatment format would be adequate for the person in question. Additionally, the number of questionnaires used in this study was similar to previous studies conducted by the same research group, in which more people applied [e.g., (42)].

The recruitment process and sample characteristics impacted our choice of design, result analyses, and possible interpretations of the results. Due to the low number of included participants (*N* = 24), we opted for a within-group design instead of randomizing the participants to a treatment group or a waitlist control group. As a result, it is more difficult to draw causal conclusions from the findings, since control over potential third-variable influences is reduced compared to when using an RCT design (88). However, the advantage of a within-group design in this study is that more participants could undergo the ICBT simultaneously, providing more information about the treatment's effectiveness at the end of the COVID-19 pandemic before it was declared over. Additionally, it can be argued that it is more ethically justifiable not to make half of the participants wait for intervention when they have applied to take part in the treatment for symptoms they are currently experiencing, even if the symptoms do not increase during that waiting time (89). The heterogeneity of the sample also influences the results and interpretation of these. There was a relatively wide range in age (20–67), employment status, and education level, which suggests that the sample is representative of the intended population, namely adults with psychological symptoms during the final phase of the COVID-19 pandemic. However, more than half (16 participants) were likely to meet the diagnostic criteria for post-COVID syndrome, while eight individuals were not assessed to meet the criteria. Of those answering the post-treatment measurement, and thus what data were used in the result analyses, two-thirds (10 of 15) of the individuals were assessed as probably having the post-COVID syndrome. Subsequently, the sample might consist of individuals from two somewhat different populations. Although no statistical tests were conducted regarding the symptom levels or changes in symptoms during the treatment, it could be hypothesized that individuals with and without post-COVID may have different needs in psychological treatment. At the same time, this may reflect individuals who experienced psychological symptoms during the final phase of the pandemic—with some with somatic and medical symptoms (90) and others without (1, 13)—even though the proportion of participants probably having post-COVID is higher in this study than in the general population. Moreover, in the present study, we were not able to definitively confirm or diagnose participants with post-COVID syndrome, which is why the term “probable” post-COVID is used. This limitation stems from the fact that we neither met participants in person nor had access to their medical records. Future studies should address this by including confirmed diagnoses when investigating differences between individuals with and without post-COVID syndrome.

### 4.1 Strengths and limitations

This study has several strengths, such as the use of standardized and well-established measures, allowing for comparison between other studies. Clinical telephone interviews for evaluation were also included, which enabled a thorough assessment. With multiple measures, we managed to get a broad picture of the participants' experienced problems and symptom change. Standard CBT strategies were used in the treatment, making it comparable to previous ICBT treatments used before and during the COVID-19 pandemic. Despite a low number of participants, decreases in psychological symptoms were identified, indicating that there is sufficient power at least to some extent.

However, a COVID-19 specific and adapted measure would have been needed to get a clearer picture of the perceived problems at the end of the pandemic. In this study, individual questions about the impact of the pandemic were used. Nevertheless, as Orsmond and Cohn (91) emphasized, new measures need to be developed alongside the evaluation of novel interventions. Moreover, one or more self-report measures related to treatment quality, such as an adapted version of the Session Evaluation Questionnaire (92) could advantageously have been used to evaluate the feasibility of the ICBT intervention over time. Such measures might have provided valuable insights into participants' experiences of the treatment and offered a more detailed understanding of potential reasons for treatment dropout. However, we conducted more in-depth interviews, including open-ended questions, after treatment conclusion with the participants who consented to be interviewed. These findings are planned to be reported in a separate article, and hopefully, these interviews will provide even richer and more nuanced information regarding participants' experiences of the ICBT intervention.

Caution is warranted when interpreting the statistical results, given the small sample size and the absence of a control condition. Additionally, the results include outliers. Furthermore, CCA was used, which means that some participants' information is lost, but at the same time, the results are based solely on actual data. With that said, this was a feasibility study whose primary aim was not to evaluate the outcome of interest but rather to investigate whether a larger, main study would be feasible and what might need to be adjusted (93). The COVID-19 pandemic specifically will (hopefully) not recur, but other types of epidemics and pandemics are likely to emerge sooner or later (94). Even research that cannot be directly generalized due to factors such as context or changes in the global situation can contribute knowledge about what might happen or what happens under exceptional circumstances. This is a study of ICBT in a context other than what has been investigated before. Hopefully, the study can be used to guide future studies within the research area, both in pandemic-related and other unique or new contexts. Practical implications based on this study include, for future research and clinical settings, an emphasis on the use of more specific measurement instruments when targeting symptoms related to a specific phenomenon or crisis (e.g., psychological symptoms related to the COVID-19 pandemic). Comparisons between treatment formats and modules featuring longer vs. shorter texts would also be valuable for the understanding of treatment effects and dropout rates. If including people with somatic symptoms to an individually tailored psychological intervention, it may be beneficial to incorporate a module or specific focus on these symptoms and their potential impact on mental health, if this is not addressed throughout the treatment. Most importantly, however, the findings suggest that individually tailored ICBT with support by a therapist appears to be a feasible and acceptable treatment option, even in the later phase of a global crisis such a pandemic, indicating ICBT as a valuable clinical tool during times of societal crisis when flexible and scalable mental health interventions are urgently needed.

### 4.2 Future research

The results of this feasibility study indicate that ICBT could be an adequate treatment option at the end of the COVID-19 pandemic. However, larger studies with more participants and in multiple countries are needed. At the time of writing, the COVID-19 pandemic has been declared over, but that does not mean its impact is. Further research on how people, with and without post-COVID syndrome, are affected and how they can be helped is needed. It would also be valuable to investigate individuals' transition from a pandemic-related to a post-pandemic society, what that process entails, and what difficulties might be encountered. Since it was not the study's aim to investigate differences between those likely to have post-COVID syndrome and those who do not, and because of the low number of participants, this was not investigated further. However, potential differences in psychological symptoms and how psychological treatments such as ICBT are received would need to be further explored.

Results from several studies indicate a correlation between post-COVID syndrome and mental health issues such as depression and anxiety (90, 95), but also cognitive impairments, such as in attention and memory (96). Thus, there is a need for psychological interventions. Even though ICBT has been reported effective for psychological symptoms related to somatic conditions, the exhaustion, fatigue, and brain fog that post-COVID syndrome can entail may make it difficult for these individuals to benefit from the treatment (97). The results in this study indicate ICBT in total to be feasible, and most of the participants who completed the post-treatment measurement probably had post-COVID syndrome. However, the small sample size and the absence of a control condition make it difficult to draw any far-reaching, definitive conclusions. Thus, further research is needed to investigate the effectiveness of ICBT for those with post-COVID syndrome.

### 4.3 Conclusions

The aim of this study was to investigate whether ICBT could be a feasible treatment for psychological symptoms during the final phase of the COVID-19 pandemic. When pre- and post-treatment measures were compared using CCA, significant decreases were observed in symptoms of depression, anxiety, and secondary outcome measures such as loneliness. However, estimates for other secondary outcome measures did not show statistically significant differences. On average, participants completed nearly half (3.96) of the eight treatment modules during the treatment period. The dropout rate was 37.5%, with three (12.5%) participants explicitly stating they wanted to quit their participation, mainly due to the excessive amount of text in the treatment program. Effort should continue to investigate the effects of ICBT and how these can be enhanced and expanded. More specifically, our results indicate that ICBT in many ways is feasible and effective for psychological symptoms during the last phase of a pandemic. However, improvements are both possible and necessary to meet the needs of even more individuals.

## Data Availability

The raw data supporting the conclusions of this article will be made available by the authors, without undue reservation.
