# Supplementary material for: Internet-based cognitive behavioral therapy for psychological symptoms during the final phase of the COVID-19 pandemic: a feasibility study
Source: Front Digit Health. 2025 Jun 9;7:1504217. doi: 10.3389/fdgth.2025.1504217 (PMC12183213; doi:10.3389/fdgth.2025.1504217)
Supplement: Supplementary file 1 [file Datasheet1.pdf]

Supplementary Material

Supplementary Material A

Semi-structured clinical interview that was conducted by telephone with the individuals who had applied for the study.

Hi!

My name is (the name of the interviewer), and I call from the PostCoronaCope study. We are now interviewing people who have applied for the study to investigate whether the treatment included in this study probably matches the specific problems you are applying for. Would you possibly have time for this now, or would you like to make an appointment for the interview?

Which current psychological symptoms have arisen or worsened in conjunction with the COVID-19 pandemic that you would like help with by participating in the study?

How does this or these problems manifest in your life?

How does this affect your daily life?

Have you been vaccinated for the SARS-CoV-2 virus? If yes: How many times? Have you experienced any side effects or been otherwise affected by the vaccine?

(Questions about different psychological problem areas. Depending on the answer, different follow-up questions were added.)

Do you have problems with sadness or lack of interest in things you used to enjoy doing?

Do you have problems with anxiety or worry?

Do you have problems with intense fear that suddenly appears, known as panic attacks?

Do you have problems with your sleep?

Do you have problems with tiredness or fatigue during the day?

Do you have problems concentrating or difficulty remembering things in a way you have not had before?

Do you have problems with stress?

Do you have problems managing difficult emotions?

Do you feel that you have problems with high demands on yourself?

Would you say that loneliness is a problem for you? Would you say that your feelings of loneliness have changed with the pandemic?

Have you experienced any or several difficult events in life that negatively affect you today? Do you think about them even though you try to avoid them?

Are you bothered by fear of infection or diseases, or suspecting you are sick?

Has your financial situation been affected by the pandemic? In what way?

Are there any obstacles or complicating circumstances you can identify for participating in the study and treatment?

Are you currently taking any medication or undergoing any (psychological) treatment?

Is there anything else that I should have asked about, or is there something else that you would like us to know about you?

That was my last question! Thank you so much for your time and answers. We will contact you soon whether you have been included in the study or not.

Supplementary Material B

Questions regarding earlier experience with psychological treatment, included in the pre-treatment measurement.

Earlier experience with psychological treatment

- Do you have any previous experience of psychological treatment (e.g. counselor, therapist, or any type of talk therapy)?
    - No
    - Yes (completed)
    - Yes (ongoing)
- If yes, what type?
- What was the focus of the treatment(s)?
- Were there any specific techniques or strategies used?
- What did you think about the treatment(s)? And why?
- Are there any of the tools or strategies described below that you have encountered before, and if so, what did you think of them?
- Have you worked on scheduling activities that are perceived as positive and that you have tried to carry out regardless of whether you felt like doing them at the moment? (sometimes referred to as behavioral activation)
    - Yes
    - No
- If yes, how was it for you to work with that?
- Have you worked on different ways to adopt new attitudes towards thoughts, especially those thoughts that negatively affect you? (sometimes referred to as cognitive restructuring)
    - Yes
    - No
- If yes, how was it for you to work with that?
- Have you gradually approached things or situations that you previously avoided? (sometimes referred to as exposure)
    - Yes
    - No

- If yes, how was it for you to work with that?
- Have you tried testing different strategies to improve your sleep, such as keeping a sleep diary or developing sleep routines?
    - Yes
    - No
- If yes, how was it for you to work with that?
- Have you worked on identifying and articulating feelings in a structured manner and tried different ways to manage those feelings? (which can be called emotion regulation)
    - Yes
    - No
- If yes, how was it for you to work with that?
- Have you worked on allowing difficult thoughts and feelings to exist when they cannot be made to disappear? That is, working on accepting them?
    - Yes
    - No
- If yes, how was it for you to work with that?
- Have you worked on mapping out which activities are stressful and which are restorative for you? That is, have you worked on stress management?
    - Yes
    - No
- If yes, how was it for you to work with that?
- Have you worked on relaxation in a structured manner, such as practicing relaxation exercises to unwind?
    - Yes
    - No
- If yes, how was it for you to work with that?
- Have you worked on tackling a problem in a structured way by brainstorming several solutions and then choosing one to test?
    - Yes
    - No
- If yes, how was it for you to work with that?

- Other tools or strategies within talk therapy that you have encountered before and are not described in the list above:

Supplementary Material C

Knowledge test about CBT, included in the pre- and post-treatment measurement.

Below are 16 questions about your knowledge of cognitive behavioral therapy (CBT). Based on what you currently know about CBT, answer the questions and indicate how confident you are in your answers, whether you are guessing, fairly sure, or entirely sure.

Choose the option that you believe is most correct according to CBT principles. Different answers may work differently depending on the individual and the situation. However, we are interested in what you believe is most important within CBT. You do not need to answer correctly, we are interested in your current level of knowledge.

- 1. According to CBT, what happens if you avoid things that evoke unpleasant feelings?
    - (0) The unpleasant feelings will gradually disappear over time.
    - (0) The unpleasant feelings can temporarily become worse.
    - (1) The unpleasant feelings will persist in the long term.
- How confident are you in your answer?
    - (1) I am guessing.
    - (2) I am fairly sure.
    - (3) I am entirely sure.
- 2. According to CBT, what happens if you avoid a harmless situation that triggers anxiety?
    - (0) It is a quick and helpful way to reduce anxiety over time.
    - (1) It is a quick way to reduce anxiety, but the anxiety will come back in similar situations.
    - (0) The anxiety increases momentarily but gradually disappears over time.
- How confident are you in your answer?
    - (1) I am guessing.
    - (2) I am fairly sure.
    - (3) I am entirely sure.
- 3. According to CBT, what is the most helpful way to manage negative thoughts?

    - (0) Try to think positively as often as possible.
    - (0) Try to ignore negative thoughts and distract yourself with something else.
    - (1) Try to allow negative thoughts to arise but challenge them.
- How confident are you in your answer?
    - (1) I am guessing.
    - (2) I am fairly sure.
    - (3) I am entirely sure.
- 4. Can anxiety be dangerous?
    - (1) No, it is not dangerous but can feel very uncomfortable.
    - (0) Yes, it is dangerous as it can lead to heart attacks or strokes.
    - (0) No, it is not dangerous, as long as it does not become too intense.
- How confident are you in your answer?
    - (1) I am guessing.
    - (2) I am fairly sure.
    - (3) I am entirely sure.
- 5. What are more effective ways to manage depression and tiredness in the long term?
    - (0) Rest properly and try to sleep as much as possible.
    - (0) Try to think more positively to avoid getting stuck in negative thoughts.
    - (1) Try to engage in activities you genuinely enjoy regardless of how you feel.
- How confident are you in your answer?
    - (1) I am guessing.
    - (2) I am fairly sure.
    - (3) I am entirely sure.
- 6. Emma says that she has low self-esteem, which prevents her from socializing more with her friends and starting with theater. What advice would CBT give her?
    - (1) She needs to try to challenge her low self-esteem and attend theater classes anyway.
    - (0) She needs to try to strengthen herself by acknowledging that she is indeed good. If she can understand that she is so good, she can start theater without feeling so anxious.
    - (0) She needs to start trying to accept that self-esteem is a kind of personality trait and is difficult to change. Perhaps she can do something other than theater.

- How confident are you in your answer?
  - (1) I am guessing.
  - (2) I am failry sure.
  - (3) I am entirely sure.
- 7. Molly fears being in the city when it is crowded. What should she try to do according to CBT?
  - (1) Gradually try to challenge the fear and be in the city anyway.
  - (0) Call someone who talks to her and calms her down while she runs errands.
  - (0) Try to hurry while thinking about something else so that the situation passes quickly.
- How confident are you in your answer?
  - (1) I am guessing.
  - (2) I am fairly sure.
  - (3) I am entirely sure.
- 8. Marcus is about to arrive at an important course but becomes nervous and experiences palpitations and chills. What can he try to do, according to CBT?
  - (0) Skip the course and go home to prevent his anxiety from getting worse.
  - (0) Try to think about something else to calm his body down.
  - (1) Try to challenge the anxiety and attend the course even though it feels very uncomfortable.
- How confident are you in your answer?
  - (1) I am guessing.
  - (2) I am fairly sure.
  - (3) I am entirely sure.
- 9. Alice finds having lunch with her colleagues in the staff room uncomfortable, so she prefers to eat alone. According to CBT, what could she try?
  - (0) Give it some time and wait until it feels comfortable to have lunch with them.
  - (1) Try challenging the discomfort and starting to eat lunch with them anyway.
  - (0) Be a little kind to herself and ignore having lunch with them if it still feels uncomfortable.
- How confident are you in your answer?

    - (1) I am guessing.
    - (2) I am fairly sure.
    - (3) I am entirely sure.
- 10. Peter is tired even though he sleeps a lot. He does not have the energy to do things he used to enjoy. According to CBT, what would be most helpful for him?
    - (0) Make sure to rest appropriately so he gradually becomes more energetic.
    - (1) Gradually start doing something he used to enjoy, even if he is tired.
    - (0) Gradually try to think more positively so that he feels a bit happier.
- How confident are you in your answer?
    - (1) I am guessing.
    - (2) I am fairly sure.
    - (3) I am entirely sure.
- 11. Edvin fears embarrassing himself in front of his friends and often declines when they want to do things together, even though he wants to. What should he try to do, according to CBT?
    - (0) Try to think away the fear and calm down by reminding himself that they are still his friends
    - (0) Try to find other activities to do where he does not have to feel anxious.
    - (1) Try to go against the fear and join them anyway.
- How confident are you in your answer?
    - (1) I am guessing.
    - (2) I am fairly sure.
    - (3) I am entirely sure.
- 12. Johan walks into a store and experiences a sudden feeling of discomfort, noticing that his heart is beating rapidly. This often happens when he is among other people. According to CBT, which strategy could be helpful to try?
    - (1) Try to act against the anxiety and continue shopping as planned.
    - (0) Try to leave the store as quickly as possible so that his body calms down.
    - (0) Try to ignore how his body feels and rush through the shopping.
- How confident are you in your answer?
    - (1) I am guessing.
    - (2) I am fairly sure.

  - (3) I am entirely sure.
- 13. Sofia is at a party and feels socially excluded. She thinks nobody likes her and that her friends find her boring. According to CBT, what is the most helpful thing for her to do?
  - (0) Try to think more positively, for example, that she is a good friend and that the party is fun.
  - (0) Try to go home so that she does not have to feel socially excluded and boring.
  - (1) Try to let the thoughts be and shift focus to what is actually happening around her.
- How confident are you in your answer?
  - (1) I am guessing.
  - (2) I am fairly sure.
  - (3) I am entirely sure.
- 14. Anna is supposed to give a presentation at work but becomes very nervous and plans to pretend to have a sore throat to avoid doing it. How does one reason, according to CBT?
  - (0) It would be good if she did not have to give the presentation because the situation is so difficult for her.
  - (0) It is an okay lie in this case because Anna has anxiety issues and may need peace and quiet.
  - (1) If Anna does not attempt to give the presentation, it might prevent her from realizing that the situation may not be as dangerous as she thinks.
- How confident are you in your answer?
  - (1) I am guessing.
  - (2) I am fairly sure.
  - (3) I am entirely sure.
- 15. Mikael is often feeling down and depressed. According to CBT, what is the most effective thing he can do to feel better?
  - (1) Try to engage in activities he used to enjoy, even if it does not feel enjoyable at the moment.
  - (0) Try only to do what he feels like doing at the moment.
  - (0) Try to stay at home, sleep, and wait out the depression.
- How confident are you in your answer?
  - (1) I am guessing.

    - (2) I am fairly sure.
    - (3) I am entirely sure.
- 16. Sara worries about her future. She often wonders what to do and gets stuck searching the web for different education and travel options. According to CBT, what would one recommend she do?
    - (0) Try to think that everything will be okay.
    - (0) Try to talk about her worries with many people until she feels calm.
    - (1) Try to pause and shift focus to what is happening within her at the moment instead of searching the web.
- How confident are you in your answer?
    - (1) I am guessing.
    - (2) I am fairly sure.
    - (3) I am entirely sure.

Supplementary Material D

Description of the modules in the individually tailored internet-based cognitive behavioral therapy.

Introduction

The introduction module includes psychoeducation about the impact of the COVID-19 pandemic on mental well-being. Information is also provided about CBT, and the participant can read about and try to understand their symptoms based on functional analysis. Furthermore, the module includes exercises identifying one's own values and treatment goals. In the end, the participant gets to read introductions to the other modules and has the opportunity to wish for which ones they would like to be involved in their treatment plan.

Behavioral activation

In this module, the participant is given the opportunity to investigate the connection between activities and well-being. The module includes psychoeducation about the importance of engaging in activities that bring positive emotions and energy into one's life. The exercises prompt the participant to reflect on their positive activities, create strategies to increase the likelihood of engaging in these activities, and, in the end, design and evaluate one's own activity plan.

Cognitive restructuring

This module introduces how different interpretations affect how individuals react to a particular situation. Negative automatic thoughts are described, as well as their relation to our well-being. The exercises involve trying to notice one's negative automatic thoughts in various situations. Based on different strategies, the participant begins to practice challenging these thoughts and then formulate new alternative thoughts.

Acceptance

In the module about acceptance, the participant receives a rationale for acceptance as an approach to dealing with challenging thoughts and emotions. The aim is for the participant to understand that the more we struggle against discomfort, the more present the discomfort will be. The exercises involve

reflecting on past approaches to managing discomfort and whether these have been helpful. Through an audio file, the participant can practice observing their thoughts and feelings. Concludingly, the participant gets to reflect on their values as a continuation of the work he/she did in the first module, Introduction.

Emotion regulation

In this module, different types of emotions and their respective functions are described. Primary and secondary emotions are highlighted. It is explained when and why emotions can often become problematic, and the participant gets to read about possible ways to manage these situations. Exercises encourage the participant to try to distinguish between primary and secondary emotions. Also, the participant gets to gradually start to label his/her own emotions and, without avoiding them, stay with the feeling to begin experimenting with acting contrary to the initial impulse.

Anxiety and exposure

This module involves psychoeducation about anxiety, including how it manifests physically and a rationale about the anxiety curve. It also explains what exposure is and how it is carried out. The participant reflects on the safety behaviors he/she uses, begins to outline an anxiety hierarchy, and, based on the anxiety hierarchy, creates a plan for exposure.

Anxiety and worry

The module starts with psychoeducation about anxiety and worry. Avoidance behaviors and physical reactions to anxiety are highlighted. Furthermore, an exposure rationale is given. The module primarily focuses on worry, its relation to well-being, and how it is maintained. In the exercises, the participant begins with mapping out his/her own worries. Afterward, the participant is encouraged to limit the worrying by saving worries for a scheduled worry time and then gets to evaluate how it went.

Social anxiety

The module about social anxiety focuses on anxiety and fear in various social situations. The module includes psychoeducation about what social anxiety is and how anxiety arises. Exercises involves describing one’s own safety and avoidance behaviors, creating an anxiety hierarchy, and then exposing oneself to the situations labelled in the hierarchy.

Panic

At the beginning of this module, psychoeducation is provided about anxiety and panic attacks. The exercises include interoceptive exposure, i.e. gradually approaching bodily sensations in different ways, such as breathing through a straw. The participant also gets to try a breathing strategy to counteract hyperventilation.

Sleep

The participant explores behaviors that can positively and negatively affect sleep. Psychoeducation about sleep patterns and sleep needs, as well as common causes of sleep problems, is also given. Sleep strategies that can lead to better sleep at night are presented, and then the participant gets to select one or two strategies to practice.

Perfectionism

This module includes psychoeducation about unhelpful perfectionism, how it is maintained, and the consequences it brings. Exercises focus on understanding one's perfectionism and offer strategies to break unhelpful thought and behavior patterns. The participant gets to practice self-compassion by writing a letter to a friend and a letter to oneself.

Stress management

The stress management module aims to teach the participant about stress and its consequences. The participant is encouraged to think of recuperative activities and plan their time to gain a better overview of his/her days. Hopefully, this will enable a reprioritization, including managing time more effectively and prioritizing the carrying out of recuperative activities.

Relaxation

The participant receives psychoeducation about the method of applied relaxation and gets to try the first part of the technique. The participant can also access an audio file with instructions for practicing muscle control in the module. Information about the remaining steps in applied relaxation is also included, and the participant is encouraged to continue practicing.

Problem-solving

In this module, the participant learns a technique for practical problem-solving. The participant formulates a problem and brainstorms several different alternative ways to address it. In the next step, the participant selects one alternative and plans how and when to implement it. Finally, the participant is encouraged to test the chosen alternative and evaluate its effectiveness for the defined problem.

Difficult memories

The module about difficult memories includes psychoeducation about how distressing events and memories can be challenging to let go of and live with. The participant learns about what happens in the body during a traumatic event and in what ways it can affect afterward. The exercises involve reflection about how the participant relates to his/her experiences, writing down the memory of an experience, and repeatedly exposing oneself to the text. Additionally, the participant gets to try two different strategies for managing difficult memories.

Focus and concentration

Psychoeducation about multitasking, attentional capacity, and concentration is provided. The module provides advice and information about breaks, structure, and environmental factors that make concentration easier. One of the exercises involves briefly shifting focus between two stimuli, and another involves practicing some of the advice presented in the module while performing a concentration-demanding task.

Manage financial stress

This module aims to give the participant strategies for managing financial stress and teach him/her how financial stress can affect his/her psychological well-being. Exercises involve exposure to thoughts and feelings about finances. The participant also gets to engage in problem-solving and acceptance activities, focusing on financial thoughts and feelings.

Loneliness

The participant receives psychoeducation about loneliness and its consequences for our well-being, along with the opportunity to map out the participant’s feelings of loneliness and what might cause them. Depending on the reason for the perceived loneliness, the participant is then offered a choice

between conducting an exercise in behavioral activation, behavioral experiment, or cognitive restructuring.

Healthy self-assertion

In this module, psychoeducation is provided about the meaning of healthy assertiveness and why, in many situations, it is a better approach than passivity or aggression. The participant also learns about a strategy for formulating one's own feelings and thoughts and is provided with examples of how to say no and set boundaries. Concludingly, the participant is encouraged to practice acting more in line with healthy assertiveness in situations where he/she previously acted passively or aggressively.

Conclusion and Maintenance plan

The conclusion and maintenance plan module focuses on the importance of continued practice and work to maintain progress made during the treatment. It emphasizes the meaning of being able to manage setbacks and unexpected events. With exercises, the participant reflects on his/her warning signs and how setbacks can be prevented and managed, and creates a maintenance plan. Also, the participant gets to reflect on what he/she takes with him/her from each module and think about whether his/her earlier defined treatment goals are still relevant, should be revised, or if new ones should be created.
